# The Sensory Quality and the Physical Properties of Functional Green Tea-Infused Yoghurt with Inulin

**DOI:** 10.3390/foods11040566

**Published:** 2022-02-16

**Authors:** Katarzyna Świąder, Anna Florowska

**Affiliations:** 1 Department of Functional and Organic Food, Institute of Human Nutrition Sciences, Warsaw University of Life Sciences (SGGW–WULS), 159C Nowoursynowska Street, 02-787 Warsaw, Poland; 2 Department of Food Technology and Assessment, Institute of Food Science, Warsaw University of Life Sciences (SGGW–WULS), 159C Nowoursynowska Street, 02-787 Warsaw, Poland; anna_florowska@sggw.edu.pl

**Keywords:** yoghurt, green tea, inulin, functional product, sensory quality, physical properties

## Abstract

The purpose of this study was to investigate the influence of the addition of inulin (3%, 6% and 9%) to green tea-infused set type yoghurt on its sensory quality and physical properties. Yogurts were made by combining green tea with milk and inulin and inoculated with freeze-dried starter cultures YO-122. Incubation was conducted at 43 °C for approximately 4.5 h until a pH value of 4.5–4.6 was achieved. For the prepared yoghurts, a panel of experts (*n* = 10) was selected, characterized 35 attributes and conducted a sensory quality assessment of these yoghurts using the Quantitative Descriptive Profile method. Additionally, instrumental analyses such as yield stress, adhesiveness, firmness, physical stability and color parameters were also carried out. The use of green tea infusion increased the perception of green tea flavor, bitterness, astringency, dark color of the yoghurt and the existing whey, which worsened the overall sensory quality of the yoghurt. The addition of inulin (9%) to the green tea yoghurt, increased the perception of sweet, peach flavor and aroma and improved the firmness of the yoghurt while reducing the perception of sour taste, which improved the sensory quality of the yoghurt. Both inulin and green tea affected the physical properties of the yoghurts, causing an increase in the yield stress (43%, and 20%, respectively) and deteriorated the stability of the yoghurts. Green tea affected the color of the yoghurts, causing the lightness to decrease. The L* parameter decreased from 89.80 for the control sample to 84.42 for the green tea infused yoghurt. The use of infused green tea in yoghurt production makes it necessary to use ingredients that will neutralize its adverse effects on sensory quality and physical parameters of yoghurt, and such an additive can be prebiotic fiber–inulin at a concentration of 9%.

## 1. Introduction

One of the most innovative food sectors in Europe is the dairy industry and it is trying to respond to consumer demands by improving its products, introducing new product formulations or technologies [1,2]. As a result, the products on offer are not only nutritious but also contain active substances which have an impact on health aspects and can be classified as functional foods. Various plant raw materials are introduced to improve the nutritional value of dairy products as well as to increase the content of substances with health-promoting properties [3]. Dairy products, including fermented drinks such as yoghurt, are a good example of this. We can find studies on enriching yoghurt with the following ingredients, among others: aloe vera gel [4], grapes [5], flaxseed [6], coconut-cake [7], pomegranate juice powder [8], dried pomegranate seeds [9], freeze-dried apple pomace powder [10], spirulina [11], saffron [12] or lotus, persimmon, rosemary, nettle, caraway, hyssop [13] and lemon balm [2,3]. The use of these ingredients can improve the health-promoting properties as well as their technological and sensory quality, although the combination of these several functions is not always possible.

Among the plant components, tea (*Camellia sinensis*) with different degrees of fermentation as green tea, white tea and black tea is increasingly appearing in studies on the production of dairy drinks with plant extracts that can be used in yoghurt to improve its antioxidant properties and maintain it during storage [13,14,15,16,17,18], as well as the influence on the overall quality; however, the studies that have been carried out so far are mainly based on hedonic tests [5,16,18,19,20].

The health benefits of yoghurt can be also obtained by using in its production plant-based ingredients with prebiotic properties, which could symbiotize with the bacteria presented in yoghurt. Inulin, which is found in high concentrations in chicory root (*Chicorium intybus*) [21] is one such example. When inulin is used in a product, it is also possible to use a nutrition claim “source or high fiber content” or a health claim if “native chicory inulin” is used in the product, stating “Chicory inulin contributes to normal bowel function by increasing stool frequency” [21]. While many studies have been conducted on the effect of inulin addition on yoghurt quality [22,23,24,25,26,27,28] to the best of our knowledge no specific studies have been conducted on the effect of both infused green tea and inulin addition on sensory quality evaluated by a panel of experts and instrumentally. The analysis of this data is of great importance in the development of new products, which translates into their purchase choice and acceptance by consumers. By using tea addition and infusion technology we can achieve a natural functional food, while the use of inulin as a prebiotic can increase both nutritional value by increasing the fiber content and the health promoting value. However, the question arises whether the product will also be attractive from a sensory point of view. Therefore, the main objective of our project process was to investigate the influence of the addition of inulin to green tea-infused yoghurt on its sensory quality assessed by a panel of experts and physical properties tested instrumentally.

## 2. Materials and Methods

### 2.1. Materials

Yoghurts were made with 3.2% fat content cow’s milk, pasteurized and microfiltered (Piątnica, Poland). Milk was inoculated with freeze-dried starter cultures YO-122 (Serowar, Poland), containing Streptococcus salivarius subsp. thermophilus and Lactobacillus delbrueckii subsp. bulgaricus. In addition, in production of yoghurt with infused tea the leaf green tea (Camellia sinensis, BioFix, Tuszyn, Poland) was used. As a prebiotic ingredient Frutafit^®^ CLR inulin (chicory root, inulin ≥85% dm, DP 2-10, sweetness 30%) (Sensus, Roosendaal, The Netherlands) was used to enrich both natural and green tea infused yoghurt.

#### Yoghurt Processing

The production process of natural and infused green tea yoghurt was carried out according to the methodology described by Świąder et al. [29]. Milk for yoghurt production with tea was first heated for 30 min to 85 °C. Then, green tea leaves (2 g tea/100 mL milk) were infused over the milk and steeped covered for 10 min. The resulting infusion was then filtered manually through gauze strainers and cooled. The infusion prepared in this way was inoculated with starter cultures (0.1%) and gently stirred and then bottled into 100 mL sterile, plastic containers with lids. The next step was incubation of yoghurts in an incubator (INE 500, Memmert, Schwabach, Germany) at 43 °C for about 4.5 h until a pH value of 4.5–4.6 was reached (Voltcraft PH-100ATC, Conrad Electronic Sp. z.o.o., Wrocław, Poland). The yoghurt samples were then cooled and stored at 4 °C until the structure was built. After this time, the samples were ready for sensory and instrumental evaluation. In contrast, for natural yoghurt, the difference was that no tea was added. In order to enrich the yoghurt with inulin, inulin was added to the milk at 3%, 6% and 9% levels before the heating process. An analytical balance (PS 1000/C/2, Radwag, Radom, Poland) was taken to weigh all the raw materials used to make the yoghurt. The yoghurt samples were coded as follows: control sample (C), control yoghurt with 6% inulin (C1), yoghurt with green tea (G), yoghurt with green tea and 3% inulin (G1), yoghurt with green tea and 6% inulin (G2), yoghurt with green tea and 9% inulin (G3).

### 2.2. Methods

#### 2.2.1. Sensory Evaluation

##### The Method

The sensory quality assessment of the produced yoghurts was carried out in accordance with the procedure described in the ISO 13299:2016 standard [30] using the Quantitative Descriptive Profile (QDP) method. The expert team selected for evaluation and defined 35 discriminators characterizing the appearance, smell, texture and taste of the evaluated samples (Table S1). Seven factors described the appearance of the samples: presence of whey, shine of surface, color intensity, adhesiveness, visual smoothness, filling the teaspoon, uniformity of consistency. Nine distinctions described the odor of the samples: sour, sweet, yoghurt, milk, fat, green tea, nectar, peach and citrus. Another seven described texture and consistency: melting, thickness in the mouth, yield stress, firmness, fat film, smoothness in the mouth, creaminess. The largest number of attributes (10) was chosen to describe the taste and flavor of the yoghurts. The taste attributes were sweet, bitter, sour, astringent, and flavor attributes were yoghurt, milky, quark, peach, green tea, nectar. In addition, body and overall sensory quality were assessed. Yoghurt aroma was first assessed by slightly tilting the lid of the yoghurt package. Then, after opening the package, the general appearance of the yoghurt was evaluated among others by dipping a spoon and observing how the yoghurt looks on the spoon. After that, the consistency of the yoghurt samples was evaluated in the mouth as well as their taste and flavor, body and overall sensory quality. The intensity of sensory attributes was evaluated by an expert panel using a 10-point unstructured linear scale (c.u.—contractual units) with extremes ranging from 0 (low perception) to 10 (high perception).

##### Expert Panel

The Quantitative Descriptive Profile assessment was carried out by a panel of 10 trained experts who met the requirements of ISO 8586:2012 [31]. They were research and teaching staff from the Institute of Human Nutrition Sciences, women aged between 35 and 53, with experience in profile assessment and yoghurt evaluation.

##### Study Conditions

The study was conducted by a panel of experts in an accredited sensory laboratory (accreditation number AB 564) meeting the requirements of ISO 8589:2007/AMD 1:2014 [32]. Evaluation of yoghurts took place in individual test booths equipped with the ANALSENS computer system (Cogitos, Sopot, Poland), which enables test planning, product evaluation and data collection. Lighting, temperature and humidity were controlled during evaluation. The evaluations were carried out in two sessions with a break of 3 h during the day.

##### Preparation and Presentation of Samples

Samples for evaluations were prepared in cylindrical, transparent, plastic containers coded with three-digit codes generated by the computer system (height, 50 mm; ø, 50 mm; volume, 100 mL). The yoghurt samples prepared in this way were randomly placed on a tray and given at 7 °C to experts who evaluated them directly from the containers. To neutralize the mouthfeel between samples, still mineral water was used.

#### 2.2.2. Instrumental Analysis

##### Yield Stress

The yoghurt’s yield stress (Pa) was analyzed by the rheometer (DV3T, Brookfield, Middleboro, MA, USA), using a vane four knife spindles V74 with a torque range HA that was constantly share with the rate 0.1 s^−1^. The presented values are the averages of six replicates. The yield stress values were analyzed using the dedicated software (PG Flash, Brookfield, Middleboro, MA, USA).

##### Textural Properties

The firmness (N) and adhesiveness (Ns) of yoghurts were measured using TA.XT Plus (Stable Microsystems, Surrey, UK) with a 5 kg load cell. The device was equipped with a cylindrical container with a 0.5-cm diameter (P/0.5R) probe. The probe was penetrating the yoghurt sample for 8 cm distance, with the speed of 1.0 mm/s, and the trigger force used was 0.01 N.

##### Physical Stability—CSA Method

The physical stability of yoghurts has been presented as a space and time related transmission profiles using LUMiSizer 6120-75 (L.U.M. GmbH, Berlin, Germany). The applied measuring setting were: wavelength 870 nm, volume 1.8 mL of dispersion; light factor: 1; 1500 rpm; experiment time, 15 h 10 min; interval time 210 s. The instability analysis that allowed to calculate the instability index was performed using the SepView 6.0; LUM (Berlin, Germany) software. The trait was quantified by dividing the sample clarification of at a given separation time by the maximum sample clarification. It is set that the instability index can take values in the range from 0 to 1, in this calculation 0 indicates a stable system whereas 1 an unstable system [33].

##### Color Parameters

The L*, a*, and b* color parameters (CIEL*a*b*) of yoghurts were analyzed with a Minolta CR-200 colorimeter (Minolta, Osaka, Japan; source of light D65, a measuring hole of 8 mm) at the surface of yoghurt. To determine color differences, total color differences were determined between yoghurts with tea added and the control sample without tea and inulin, and between yoghurts with green tea and inulin added and the control sample with inulin added. The total color difference (Δ*E*) was calculated [34]:

$$\Delta E=\sqrt{{\left(L_{C}^{*}-L_{G}^{*}\right)}^{2}+{\left(a_{C}^{*}-a_{G}^{*}\right)}^{2}+{\left(b_{C}^{*}-b_{G}^{*}\right)}^{2}}$$

where $L_{c}^{*},\;a_{c}^{*},b_{c}^{*}$ and $L_{G}^{*},\;a_{G}^{*},b_{G}^{*}$ refers to the color parameters of compared yoghurts *C* is a control sample and *G* is a yoghurt with green tea addition.

Depending on the Δ*E* calculated values the color difference between the yoghurts can be estimated as not noticeable for the observer (0 < ∆*E* < 1), noticeable but only by experienced observer (1 < ∆*E* < 2), noticeable by unexperienced observer (2 < ∆*E* < 3.5), clear difference in color is noticed (3.5 < ∆*E* < 5) and observer notices two different colors (5 < ∆*E*) [34].

#### 2.2.3. Statistical Analysis

The obtained results of sensory and instrumental analysis presented on the tables and figures are the mean values with the standard deviation (±SD), data were statistically analyzed using Statistica 13.3 (TIBICO Software Inc., Palo Alto, CA, USA). To determine the significance differences in the intensiveness of the different sensory characteristics as well as the differences between the average values of yield stress, firmness, adhesiveness, instability index, and color parameters of yoghurts a one-way ANOVA analysis of variance were used. Significant differences in the intensity of sensory attributes were verified by Fisher’s post hoc NIR test at the significance level of *p* ≤ 0.05, while significant differences between yoghurts in instrumental assessment by Tukey’s test at the significance level of α = 0.05. In addition, using the built-in statistical package of XLSTATS software, Principal Components Analysis (PCA) was performed to investigate similarities and differences in the sensory quality profile of the samples.

## 3. Results

### 3.1. Sensory Evaluation

#### 3.1.1. Quantitative Descriptive Profile Analysis

The use of infused green tea as well as inulin in yoghurt production significantly influenced the sensory quality of yoghurt. Details of the research results obtained are presented in Table S2 (Supplementary Material).

##### Odor

**Figure 1:**
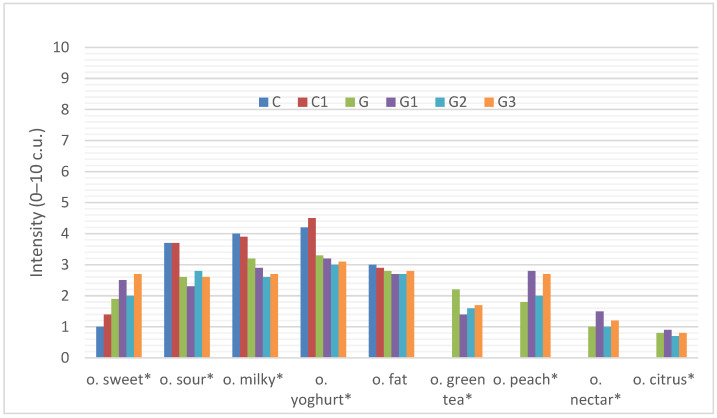
Sensory profile of the odor of yoghurts C, C1, G, G1, G2. The abbreviations in the figure refer to the control sample (C), control yoghurt with 6% inulin (C1), yoghurt with green tea (G), yoghurt with green tea and 3% inulin (G1), yoghurt with green tea and 6% inulin (G2), yoghurt with green tea and 9% inulin (G3) (o- odor; * significantly differed at *p* ≤ 0.05).

The sensory profile of the odor of six types of yoghurts is shown in Figure 1.

We can note that the addition of inulin and the use of infused green tea in the yoghurt production process significantly influenced the aroma profile of the yoghurts. The control (C) yoghurt was characterized by an intense yoghurt (4.2 c.u.), milky (4.0 c.u.), sour (3.7 c.u.), fatty (3.0 c.u.) and slightly sweet smell (1.0 c.u). The control yoghurt with inulin had a similar sensory quality of yoghurt (4.5 c.u.), milky (3.9 c.u.), sour (3.7 c.u.), fatty (2.9 c.u.) smell and was slightly sweeter in smell (1.4 c.u.) than the control sample, but in terms of yoghurt, milk, sour and fatty and sweetness smell the control yoghurt and the control sample with inulin did not differ statistically significantly from each other.

The use of infused green tea in the yoghurt changed the smell of the yoghurt. It was characterized by a light green tea infusion (2.2 c.u.) with a delicate peach (1.8 c.u.), nectar (1.0 c.u.) and citrus (0.8 c.u.) smell, which differed significantly from the control yoghurt (C) and the control with inulin (C1). Yoghurt with green tea was also significantly more intensely sweet (1.9 c.u.) in aroma than the control sample without tea and significantly less sour (2.6 c.u.), milky (3.2 c.u.) and yoghurt-like (3.3 c.u.) in odor.

On the other hand, the addition of inulin at different levels (G1, G2 and G3) to green tea-infused yoghurt did not significantly change the odor of green tea infused yoghurt (G). With the addition of inulin, the sweet and peach smell increased (G3-2.7 c.u. and 2.7 c.u., respectively) and the green tea smell decreased (1.7 c.u.), but there were no significant differences.

##### Appearance Perceived Visually

**Figure 2:**
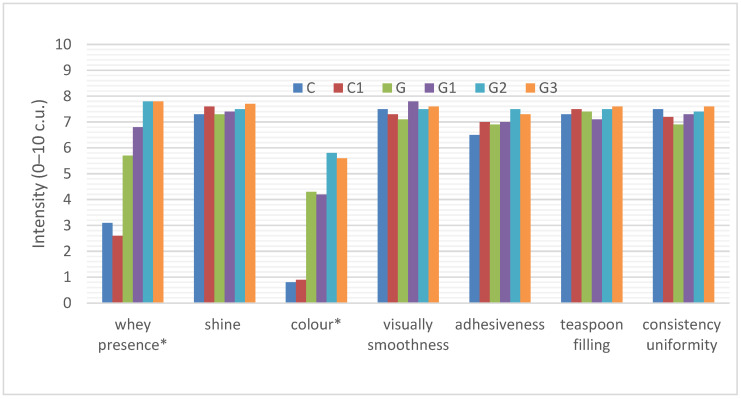
Sensory profile of the appearance of yoghurts C, C1, G, G1, G2. The abbreviations in the figure refer to the control sample (C), control yoghurt with 6% inulin (C1), yoghurt with green tea (G), yoghurt with green tea and 3% inulin (G1), yoghurt with green tea and 6% inulin (G2), yoghurt with green tea and 9% inulin (G3) (* significantly differed at *p* ≤ 0.05).

The appearance of the six yoghurts was also changed by the use of infused green tea and the addition of inulin (Figure 2).

The control sample (C) was white (0.8 c.u.) with a shiny surface (7.3 c.u.) and whey presence (3.1 c.u.). It was characterized by a visually perceptible smoothness (7.5 c.u.), adhesion to the spoon (6.5 c.u.) and filling of the spoon (7.3 c.u.) after scooping the yoghurt with a spoon as well as uniformity of consistency (7.5 c.u.). The addition of inulin to natural yoghurt had a slight effect on reducing whey flow (2.6 c.u.) and increasing adhesiveness (7.0 c.u.), but these differences were not statistically significant. Both natural (C) and natural yoghurt with inulin (C1) did not differ statistically significantly in appearance.

There was a statistically significant difference in the color and flow of whey in the yoghurt with green tea added. Yoghurt with green tea had a significantly higher whey flow compared to natural yoghurts (C and C1), as well as a significantly darker creamy-grey color (4.3 c.u.). The other appearance parameters of yoghurt did not change significantly. However, the addition of inulin to green tea yoghurt significantly increased the whey flow in this yoghurt, to 7.8 c.u. in green tea yoghurt with inulin added at 9% (G3), and the darkening of the yoghurt sample (G2—5.8 c.u.; G3—5.6 c.u.).

##### Consistency Perceived in the Mouth

**Figure 3:**
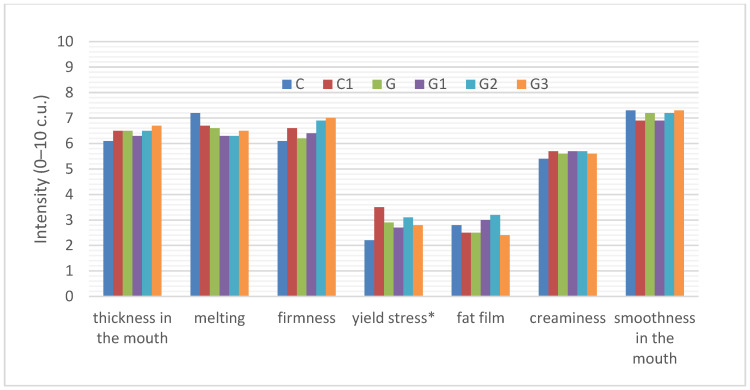
Sensory profile of the consistency of yoghurts C, C1, G, G1, G2. The abbreviations in the figure refer to the control sample (C), control yoghurt with 6% inulin (C1), yoghurt with green tea (G), yoghurt with green tea and 3% inulin (G1), yoghurt with green tea and 6% inulin (G2), yoghurt with green tea and 9% inulin (G3) (* significantly differed at *p* ≤ 0.05).

The sensory profile of six types of yoghurts consistency perceived in the mouth is shown in Figure 3. 

In the case of all the yoghurts analyzed, we can see that they were characterized by a similar consistency assessed by spreading the yoghurt samples in the mouth. All the yoghurts were thick, compact, smooth, creamy, well-melting in the mouth, slightly viscous, with a perceptible fat film. Addition of inulin had a statistically significant effect on yoghurt viscosity, causing an increase in viscosity of the control yoghurt with inulin (C1—3.5 c.u.) compared to the control yoghurt (C—2.2 c.u.). The addition of infused green tea to the yoghurt (G) slightly increased the viscosity of the yoghurt (2.9 c.u.) but there was no statistically significant difference. The addition of inulin at different levels to the yoghurt with infused green tea did not significantly change the yoghurt viscosity.

##### Flavor/Overall Quality

**Figure 4:**
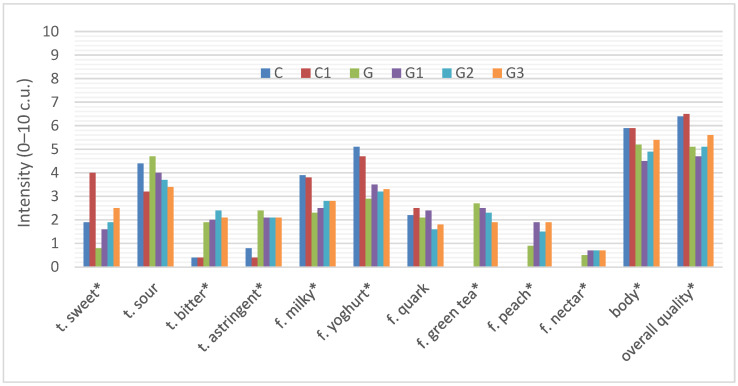
Sensory profile of the taste/flavor/overall quality of yoghurts C, C1, G, G1, G2. The abbreviations in the figure refer to the control sample (C), control yoghurt with 6% inulin (C1), yoghurt with green tea (G), yoghurt with green tea and 3% inulin (G1), yoghurt with green tea and 6% inulin (G2), yoghurt with green tea and 9% inulin (G3) (t—taste, f—flavor; * significantly differed at *p* ≤ 0.05).

The taste and flavor of the six yoghurts was changed by the use of infused green tea and the addition of inulin (Figure 4).

The control (C) yoghurt was characterized by an intense yoghurt (5.1 c.u.), milky flavor (3.9 c.u.), sour taste (4.4 c.u.), as well as slightly sweet taste (1.9 c.u) and quark flavor (2.2 c.u.), with a lightly perceptible astringent (0.8 c.u) and bitter taste (0.4 c.u). The addition of inulin to natural yoghurt significantly influenced sweet and sour taste perception. The sweet taste (4.0 c.u.) was more pronounced in the yoghurt with inulin (C1) and the sour taste was less pronounced (3.2 c.u.) than in the control natural yoghurt (C). The addition of inulin had no statistically significant effect on the other taste/flavor characteristics of the natural yoghurt.

The use of infused green tea also had a significant effect on the flavor profile of the yoghurt. The yoghurt with green tea became significantly more bitter (1.9 c.u.) and astringent (2.4 c.u.) in taste. In this yoghurt, the taste was typical for green tea (2.7 c.u.) and slight peach (0.9 c.u.) and nectar (0.5 c.u.) flavors were also significantly perceptible compared to natural yoghurt (C). Additionally, green tea yoghurt was significantly less sweet (0.8 c.u.), milky (2.3 c.u.), yoghurt-like (2.9 c.u) and quark (2.1 c.u) in taste. The addition of inulin at different levels to the green tea infused yoghurt significantly increased the sweet taste (G3—2.5 c.u.) and peach flavor (G3—1.9 c.u.) in the yoghurt, especially with the highest level of inulin and decreased the sour taste (G3—3.4 c.u.). The remaining tastes were perceived at similar levels regardless of the amount of inulin added to the yoghurt infused with green tea. 

The addition of inulin to the natural yoghurt, although increased the sweet taste and decreased the bitter taste, had no significant effect on the body and overall sensory quality of the natural yoghurt. Both yoghurts, natural (C) and natural with inulin (C1), had a similar body (C—5.9 c.u.; C1—5.9 c.u.) and overall quality (C—6.4 c.u.; C1—6.5 c.u.). 

However, significant changes in the overall quality of yoghurt resulted from the use of infused green tea in yoghurt production. The addition of green tea significantly reduced the sensory quality of the yoghurt (5.1 c.u.) and only the addition of inulin at the highest level raised the overall sensory quality of the yoghurt to 5.6 c.u.

#### 3.1.2. Principal Component Analysis

**Figure 5:**
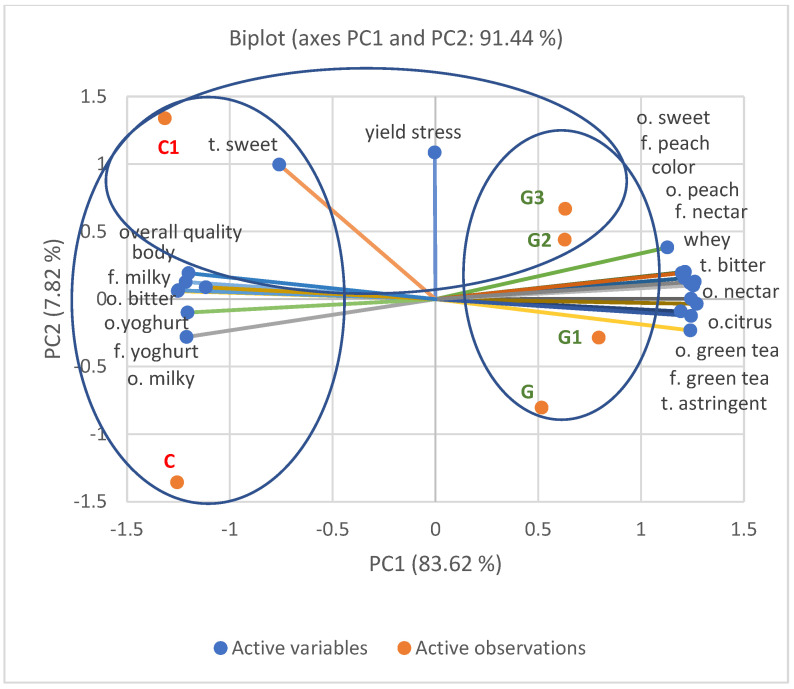
Principal Component Analysis (PCA) of the yoghurt samples: C, C1, G, G1, G2, G3. The abbreviations in the figure refer to the control sample (C), control yoghurt with 6% inulin (C1), yoghurt with green tea (G), yoghurt with green tea and 3% inulin (G1), yoghurt with green tea and 6% inulin (G2), yoghurt with green tea and 9% inulin (G3) (o—odor, t—taste, f—flavor). Attributes that significantly statistically differentiated the samples are on PCA (*p* ≤ 0.05).

Principal Component Analysis (PCA) was also performed for all yoghurt samples (C, C1, G, G1, G2, G3). In Figure 5, out of the 35 evaluated factors, only those factors are presented which, according to the statistical evaluation, significantly differentiated (*p* ≤ 0.05) the samples. In the Principal Component Analysis of the six yoghurt types analyzed, the variability of the samples was attributed to the principal component (PC1), which accounted for 83.62% of the total variability, and was assigned to viscosity (yield stress) and a second component (PC2) which accounted for 7.82% of the total variation was assigned to overall quality.

The positioning of the tested samples on the PCA graph indicates differences in their quality. The samples formed two expressive clusters. The first cluster contained samples of natural yoghurt (C) and natural yoghurt with inulin (C1), while the second cluster on the opposite side contained samples of yoghurts with infused green tea (G) and yoghurt with green tea and inulin at three different levels (G1, G2, G3).

Natural yoghurt (C) and natural yoghurt with inulin (C1) were characterized by a milky and yoghurt-like taste and smell and by a sour smell. These characteristics were positively correlated with the body and overall quality. Similar correlations were observed in the QDP analysis, where control yoghurt and natural yoghurt with inulin were characterized by high body and overall quality. On the opposite side of the PCA graph were yoghurt samples with infused green tea and with added inulin, that correlated negatively with the overall quality. 

Yoghurt samples with infused tea and with inulin added to it (G, G1, G2, G3), were correlated with green tea flavor and odor, astringent and bitter taste, peach and nectar flavor and odor, as well as citrus flavor, more intense color and whey flow. As can be seen from the QDP evaluation, characteristics such as astringent and bitter taste as well as taste and smell typical for green tea and dark color and intensive whey flow had a negative influence on the overall quality of the evaluated samples of green tea-infused yoghurt and with inulin added to it, which corresponds with the PCA results.

In addition, on the PCA graph we can distinguish another cluster which shows the influence of inulin on the quality of analyzed yoghurts. Natural yoghurt with inulin added at the level of 6% (C1) as well as yoghurt with infused green tea and inulin added at the levels of 6% (G2) and 9% (G3) were positively correlated with higher yoghurt sweetness in taste and smell as well as with higher viscosity and peach taste and smell. On the other side of the PCA graph are samples of natural yoghurt without inulin and infused with green tea and yoghurt with green tea and the lowest inulin content of 3%, which were characterized by lower viscosity (yield stress) and lower sweetness. These relationships are also reflected in the results of the QDP analysis.

### 3.2. Instrumental Analysis

**Figure 6:**
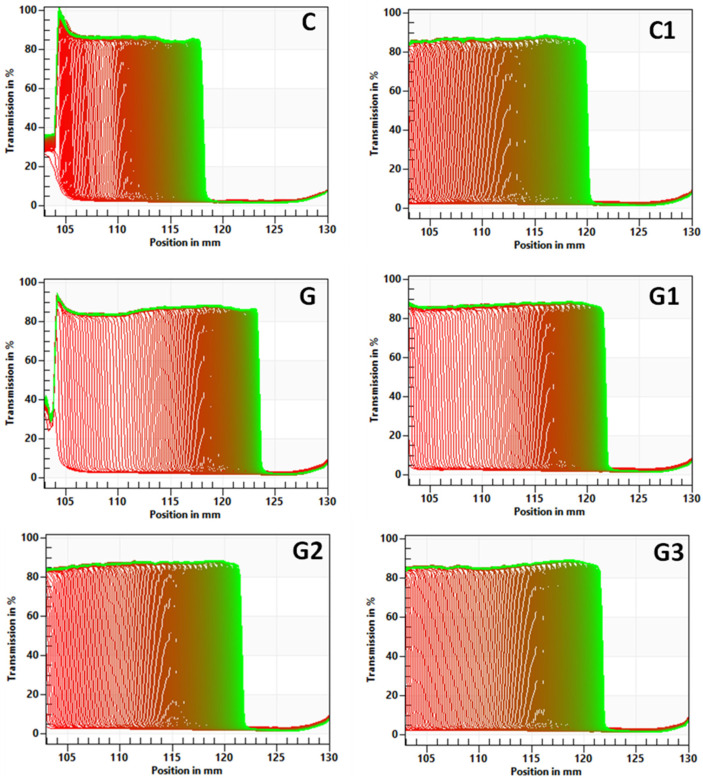
Influence of green tea and inulin addition on stability of set yoghurts indicated as transmission profiles presented enabling LUMiSizer^®^ analysis. The abbreviations in the figure refer to the control sample (C), control yoghurt with 6% inulin (C1), yoghurt with green tea (G), yoghurt with green tea and 3% inulin (G1), yoghurt with green tea and 6% inulin (G2), yoghurt with green tea and 9% inulin (G3).

**Figure 7:**
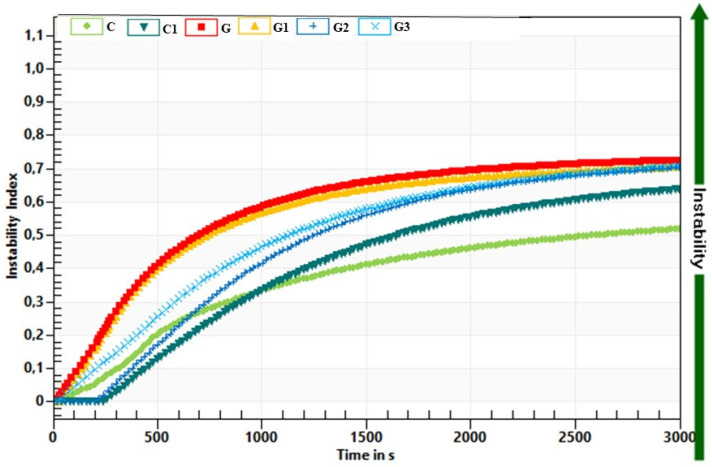
Influence of green tea and inulin addition yoghurt instability index. The abbreviations in the figure refer to the control sample (C), control yoghurt with 6% inulin (C1), yoghurt with green tea (G), yoghurt with green tea and 3% inulin (G1), yoghurt with green tea and 6% inulin (G2), yoghurt with green tea and 9% inulin (G3).

To complete the sensory evaluation of yoghurts, an instrumental analysis was also performed, the results of which are presented in Table 1 and Table 2 and Figure 6 and Figure 7, Figure S1. 

The results obtained in the measurement of the yield stress of the tested yoghurts showed that addition of inulin regardless of green tea infusion had a significant effect on this parameter.

The highest yield stress was noticed for the C1, G2 and G3 samples that contained 6 or 9% of inulin. Additionally, the green tea infusion resulted in increasing of the yield stress from 127.2 Pa obtained for the control sample to 154.9 Pa for the sample with green tea. The same tendencies were found in the firmness measurement. The greatest impact on this parameter had addition of inulin. The difference between the control samples C and C1 were significant and reached the level of 0.319 N. The highest force needed for breaking the yoghurt structure (1.302 N) was noticed for the highest tested inulin concentration (9%), whereas the lowest one (0.817 N) for the control yoghurt C. The yoghurt’s infusion did not influence the firmness of the samples. The green tea addition resulted in significant growth (more than doubled) of the adhesiveness, as compared to the control sample C1, however the inulin addition in the highest concentration diminished the green tea impact, and the adhesiveness of G3 yoghurt was similar to the control sample with inulin.

The sensory evaluated color was compared with the instrumental color measurement. The obtained results are presented in the Figure S1 and in Table 2. 

The biggest difference, which can be detected by not experienced observer, were discovered after green tea addition (Δ*E* = 8.47). The green tea addition resulted in darkening of the samples. Generally, the inulin addition did not improve the lightness of the yoghurts. The control yoghurt C and control yoghurt with inulin C1 were also not significantly differ. 

The significant difference was also noticed for evaluation a* color parameters. All tested green tea-infused yoghurts were characterized by significantly higher a* parameter, what is more the a* values of green tea yoghurts were positive what indicates that their color changed towards red color. The addition of inulin did not influence this parameter. Similar observations were made for the b* parameter. Significantly higher values of b* parameter was obtained for samples with green tea addition (b* for C—9.52, and for G—15.69) whereas the inulin addition did not influence this parameter. There were no differences between samples C and C1, but also there were no differences between yoghurt with green tea addition and different concentration of inulin (3–9%).

The stability of yoghurts was examined with the multi-sample analytical centrifuge based on the space-time resolved extinction profiles technology (STEP). The start transmission profiles of tested yoghurts were over 80% regardless of green tea addition and inulin concertation (Figure 6). 

The analysis of transmission profiles showed that on the top-part of the products the syneresis was detectable. The most stable yoghurts according to the instability index analysis were control samples, without the addition of green tea (Table 2). 

The instability index for yoghurt without green tea and inulin addition (C) was 0.517, whereas after inulin addition (C1) was 0.618. The green tea addition increased the instability index to 0.702–0.725. The separation of the fluid layer (syneresis) of yoghurts with green tea was the fastest at the beginning of the measurement, and then the greatest differences between the samples were also visible (Figure 7). 

At the end of the measurement (after 15 h), the samples had similar instability index values regardless of the inulin addition.

## 4. Discussion

The use of infused green tea in the yoghurt production process significantly influenced its sensory quality and textural properties. The same applies to the addition of inulin to the evaluated yoghurts. From previous studies we can see that the sensory quality and texture properties of the tea-infused yoghurt will depend significantly on the type of tea used [2]. Green tea is characterized by bitter and astringence taste, and floral, grassy or burn leaf flavor [35], which significantly reduced the overall sensory quality of the yoghurt infused with green tea [2]. The use of green tea in yoghurt production also significantly affected the appearance of the yoghurt, which is also confirmed in this study. The yoghurt was characterized by a large whey flow and a dark creamy color, which is also reflected in the instrumental analysis. In a study by Bulut et al. [19] on yoghurt with extracts of various plants and green tea, among others, the effect of green tea on the acceptability of yoghurt and on texture parameters was observed. Although a detailed sensory evaluation of the yoghurts with experts and the QDP method supported by the statistical analysis was not carried out in this study, the hedonic evaluation of yoghurts with green tea used by the investigators indicated the lower appearance of yoghurt with green tea extract than control one. Additionally, the lower overall score of the yoghurt with green tea extract compared to the other samples evaluated could, according to the evaluators, be attributed to the undesirable garlic and iron taste present in it. Such extraneous flavors were not perceived by the experts in the samples of yoghurt with infused green tea [2]. The study by Bulut and co-authors [19] used the addition of green tea extract to the yoghurt, while our methodology used infusion with green tea leaves, which may also have influenced the results obtained. Similar to Bulut et al. [19], a hedonic evaluation of yoghurts with green tea extract was conducted by Shokery et al. [16], who evaluated the acceptability of appearance, color, smell, flavor and overall acceptability of the yoghurt with green tea extract. This study found that yoghurt with green tea extract was rated significantly lower in appearance, odor, taste and overall acceptability than the control yoghurt, while still its acceptability ratings oscillated quite high between 5.9 and 7.2, while the control yoghurt ranged from 8.1 to 9.6 on a scale of 0–10, depending on the attribute assessed and the storage period. Acceptability ratings of appearance, taste, texture of yoghurts with green tea extract and its overall acceptability using a five-point hedonic scale were also studied by Rahmani and co-authors [20]. The results obtained by the researchers indicated that the addition of green tea extract worsened its taste, appearance, texture and overall acceptability compared to the control sample without tea, but still the yoghurt was above the acceptable level.

In our study, the addition of infused green tea to yoghurt significantly worsened the overall sensory quality of the yoghurt compared to the control sample, especially for the quality of taste and smell of the yoghurt. In order to improve the sensory quality of the infused green tea yoghurt in our study, we decided to verify the effect of the addition of inulin on its quality. 

The results show that the addition of inulin to green tea infused yoghurt increased the perception of sweet smell and taste and peach taste in yoghurt and decreased the perception of sour taste. This increased the body and overall quality of the green tea-infused yoghurt to a level similar to the overall quality and body of the control yoghurt (there was no statistically significant difference between the plain yoghurt and the green tea yoghurt with 9% inulin). This was best seen with inulin addition at the highest level (9%).

The lack of similar detailed studies on expert evaluation of sensory quality of yoghurts with infused tea and the inulin addition makes the comparison of results difficult, but on the other hand points to a new research area that we have addressed in this study. A little more research was conducted on the evaluation of textural properties of yoghurts with green tea and yoghurt with inulin, therefore we refer to them more extensively in the discussion.

The yoghurt is characterized by semisolid texture which is built from creation of a three-dimensional network mostly of milk proteins but also with the polysaccharides and fats. It is known that the main factor responsible for milk gelatinization is the reduction of the high negative net charge on casein micelles as a result of acid release from microbial activity. Thanks to the fermentation, casein micelles and denatured whey proteins, aggregate into structures through hydrophobic and electrostatic bonds, building the yoghurt structure [36]. The yoghurts tested in our experiments are set types, that form a dispersion system consisting of small particles that are responsible for the formation of yield stress. This parameter is defined as the initial force required to initiate the yoghurt to flow [37]. Both the addition of green tea and inulin into yoghurts influenced the yield stress. For the sensory evaluation, only the addition of inulin at a level of 6% increased the yield stress of the control yoghurt and with green tea.

The green tea affected the yield stress probably by the presence of polyphenolic compounds, which are able to interact with milk proteins and in consequence increasing the yield stress [38]. The same results obtained Najgebauer-Lejko et al. [39], who tested the influence of the addition of green tea water extract to yoghurt and Dönmez et al. [40] who tested effect of green tea powder addition on yoghurt structure. On the other hand, the inulin addition caused the increase of a total solids of yoghurts and probably molecules of inulin dispersed among the casein micelles interfering protein matrix formation [41]. Similar results as obtained in our work—discovering the positive influence of the inulin on the rheological parameters including yield stress—was found by Guggisberg et al. [28]. Authors have investigated the addition of 1–4% inulin into the yoghurts and discovered that yield stress values generally increased with rising levels of inulin, so it increased similar to the increase of total solids. On contrast Paseephol et al. [27] discovered that the addition of inulin (at the level of 4%) to yoghurt altered the rheological and textural properties of the product. The inulin-containing yoghurts showed a low magnitude of yield stress value and firmness than yoghurts did without inulin. 

The firmness of yoghurt is directly dependent on its total solids [36], and that is why it increase with the addition of inulin and in direct proportion to its concentration. Inulin has a gelling property. Inulin gels are composed of a tridimensional network of insoluble submicron crystalline particles that immobilized large amounts of water [42]. What is more, inulin is a water-binding agent, which is why in yoghurt it might act as a thickener by combining with the protein aggregates [25]. The network of inulin gel is an additional structure to the protein network, what results in obtaining yoghurts that requires greater strength to destroy their structure [27]. The influence of the inulin on the yoghurt firmness depends also on the chain length and the degree of inulin polymerization. In the work the long-chain inulin was used, which is why the yoghurts with inulin were characterized by higher firmness values, but on the surface of yoghurt the syneresis was observed (Figure 6) in sensory and instrumental analysis. Similar observations were made by Paseephol et al. [27].

The yoghurts with green tea were characterized by higher adhesiveness values, but no significant differences were observed in the sensory evaluation. Bulut et al. [19] fund that green tea addition increased significantly the adhesiveness of yoghurt. This effect was probably caused by the presence in green tea yoghurts polyphenolic compounds, which are known to interact with milk proteins [38]. The protein-polyphenol associates hydrogen bonds consolidated gel network, as it was stated by Harbourne et al. [43], for the acidified milk gels, fortified with gallic or tannic acid.

The instrumentally measured color parameters of the yoghurts leads to the observation that lightness (L* color parameter) was significantly affected by the green tea presents. All probes with green tea were significantly darker. This was evident in both sensory and instrumental tests. Similar founding of decreasing the L* values after addition the plant extract into yoghurts was made by Shokery et al. [16]. Authors had investigated the color changes of set-type yoghurts enriched with the extracts of green tea and moringa leaves. Additionally, Bulut et al. [19] found out that the addition of green tea extracts at 0.5% (*w*/*v*) level to yoghurts slightly lowered L* values. For the darker color of green tea yoghurts, pigments such as chlorophyll and carotenoids as well as catechins degradation products are responsible [44]. The green tea addition also influenced the a* parameter, all green tea yoghurts were characterized by the positive value of this parameter. This change is caused probably by phenolic compounds of tea, that are easily degraded by oxidative changes and form colorless or brown-colored compounds [45]. The same shifting towards positive values of a* parameter was reported by Bulut et al. [19] and Najgebauer-Lejko et al. [46]. The green tea infusion also changed the b* color component significantly. The color of the yoghurts with green tea was significantly changed towards yellowish. For this change tea colorants such as chlorophyl and its degradation as well as polyphenol autoxidation are also responsible [47].

The spontaneous whey separation of the yoghurts can result from the unstable gel network. Responsible for its formation might be the rearrangements of the gel matrix or damage to the weak gel network [48] caused by the interaction of the lactic acid produced during fermentation. Observed in our work the deterioration of stability of yoghurts after addition of green tea is a phenomenon commonly described in the literature [46,49,50]. On the other hand, Dönmez et al. [40], who tested the green tea powders addition into yoghurts reported that green tea increased the stability and caused the reduction of whey separation. The differences between the stability may be due to the different yogurt production method used compared to our experiment. Authors have explained the phenome by formation of interactions between polyphenols from tea and milk proteins. The differences between the effects of tea on the stability of the gel structure may result primarily from the differences in pH of the tested systems. According to the literature, this pH will have the greatest influence on the formation and maintenance of a stable yoghurt gel network [51]. In our experiment the addition of inulin into green tea-infused yoghurt did not change the stability. What is more, its addition to the control yoghurt without green tea also resulted in lowering the stability of the yoghurt. The effect of lower stability can be detected sensorially by evaluation of way separation, it was observed that green tea caused a greater whey separation in yoghurt, not leveled by inulin addition. 

According to literature data, inulin should act as a water holding agent and increase the stability by limiting the occurrence of syneresis [52]. Inulin being a polydisperse polysaccharide should strengthening the network and improving the whey binding capacity of the yoghurts. However, in our work we obtained different results. Additionally, Guven et al. [24] discovered that addition of inulin in ranges 1–3% did not influence the yoghurt stability. On the other hand, the deterioration of stability after inulin addition was reported by Moghadam et al. [53], where the authors explain that the results are caused by occurrence of bacterial enzymatic activity (proteolytic) that influence on the casein network. Additionally, Arango et al. [54] reported that the spontaneous syneresis increased with the inulin content in low fat yoghurts. 

The reason why the inulin did not stabilize the structure is explained by the fact that the gel structure of inulin becomes stronger and coarser with larger pores, increasing permeability and syneresis. The reason why in our experiment the inulin did not work as a stabilizer might be additionally connected with the fact that inulin properties depend on many factors such as inulin molecular weight and size, interaction with solvent, pH, temperature and process conditions [55].

## 5. Conclusions

The use of green tea infusion and inulin in yoghurt production has significantly changed the characteristics of yoghurt. Based on QDP analysis, it was found that the use of infused green tea in yoghurt production resulted in a significant increase in the perception of green tea flavor, bitterness, astringency, dark color of yoghurt and whey presence, while the perception of milky, yoghurt and sweetness decreased, which significantly worsened the overall sensory quality. On the other hand, the addition of inulin to the green tea yoghurt, especially at the level of 9%, significantly increased the perception of sweet, pleasant peach flavor and aroma and improved the firmness of the yoghurt while reducing the perception of sour taste, which improved the sensory quality of the yoghurt. The addition of green tea and inulin also affected physical parameters, which were measured instrumentally. Both additives changed the stability of the yoghurts, causing deterioration and separation of whey. Green tea significantly changed the color of the yoghurts by reducing the lightness. Green tea had a positive effect on the yield stress, the mean values increased after its addition, which was also enhanced by inulin at the highest concentration of 9%. The use of infused green tea in yoghurt production makes it necessary to use ingredients that will neutralize its adverse effects on sensory quality and physical parameters of yoghurt, and such an additive can be inulin at a concentration of 9%.

## Figures and Tables

**Table 1:** The physical properties of yoghurts. The abbreviations in the table refer to the control sample (C), control yoghurt with 6% inulin (C1), yoghurt with green tea (G), yoghurt with green tea and 3% inulin (G1), yoghurt with green tea and 6% inulin (G2), yoghurt with green tea and 9% inulin (G3).

Sample	Yield Stress [Pa]	Texture	
		Firmness [N]	Adhesiveness [Ns]
C	127.2 ^a^ ± 6.7	0.817 ^a^ ± 0.111	−0.048 ^b^ ± 0.007
C1	182.3 ^d^ ± 7.7	1.136 ^bc^ ± 0.068	−0.078 ^ab^ ± 0.009
G	154.9 ^bc^ ± 9.7	0.950 ^ab^ ± 0.105	−0.104 ^a^ ± 0.011
G1	149.5 ^ab^ ± 16.5	0.923 ^ab^ ± 0.115	−0.101 ^a^ ± 0.010
G2	176.8 ^cd^ ± 7.7	1.058 ^b^ ± 0.036	−0.105 ^a^ ± 0.028
G3	189.9 ^d^ ± 7.2	1.302 ^c^ ± 0.058	−0.087 ^ab^ ± 0.013

Values are mean ± SD (*n* = 3), a, b, c, d—values followed by the same letter within a column do not differ significantly according to Tukey’s test (*p* < 0.05).

**Table 2:** Influence of the green tea addition on color parameters and the total color difference parameter of yoghurts. The abbreviations in the table refer to the control sample (C), control yoghurt with 6% inulin (C1), yoghurt with green tea (G), yoghurt with green tea and 3% inulin (G1), yoghurt with green tea and 6% inulin (G2), yoghurt with green tea and 9% inulin (G3).

Sample	Color Parameters				Instability Index
	L*	a*	b*	Δ*E*	
C	89.80 ^c^ ± 0.59	−1.31 ^a^ ± 0.06	9.52 ^a^ ± 0.22	-	0.517 ^a^ ± 0.022
C1	90.47 ^c^ ± 0.13	−1.20 ^a^ ± 0.04	10.17 ^a^ ± 0.15	0.97 ± 0.43	0.618 ^b^ ± 0.018
G	84.42 ^b^ ± 0.19	0.78 ^b^ ± 0.21	15.69 ^b^ ± 0.77	8.47 ± 0.52	0.705 ^c^ ± 0.021
G1	85.41 ^b^ ± 0.47	0.90 ^b^ ± 0.25	15.91 ^b^ ± 0.30	1.40 ± 0.59	0.702 ^c^ ± 0.025
G2	82.35 ^a^ ± 0.10	0.97 ^b^ ± 0.05	15.15 ^b^ ± 0.07	2.24 ± 0.39	0.706 ^c^ ± 0.012
G3	82.35 ^a^ ± 0.62	1.01 ^b^ ± 0.44	15.45 ^b^ ± 0.73	2.47 ± 0.82	0.725 ^c^ ± 0.030

Values are mean ± SD (*n* = 3), a, b, c—values followed by the same letter within a column do not differ significantly according to Tukey’s test (*p* < 0.05).

## Data Availability

Data is contained within the article (or supplementary material).

## Supplementary Materials

The following supporting information can be downloaded at: https://www.mdpi.com/article/10.3390/foods11040566/s1, Table S1: Table of sensory attributes and their definitions for the profiling of yoghurts, Table S2: Results of sensory evaluation of yoghurts by panel of experts (*n* = 20). The abbreviations in the table refer to the control sample (C), control yoghurt with 6% inulin (C1), yoghurt with green tea (G), yoghurt with green tea and 3% inulin (G1), yoghurt with green tea and 6% inulin (G2), yoghurt with green tea and 9% inulin (G3) (* significantly differed at *p* ≤ 0.05), Figure S1: Color parameters in yoghurts: C, C1, G, G1, G2, G3. The abbreviations in the figure refer to the control sample (C), control yoghurt with 6% inulin (C1), yoghurt with green tea (G), yoghurt with green tea and 3% inulin (G1), yoghurt with green tea and 6% inulin (G2), yoghurt with green tea and 9% inulin (G3).
