# Supplementary material for: The Sensory Quality and the Physical Properties of Functional Green Tea-Infused Yoghurt with Inulin
Source: Foods. 2022 Feb 16;11(4):566. doi: 10.3390/foods11040566 (PMC8870793; doi:10.3390/foods11040566)
Supplement: Supplementary file 1 [file foods-11-00566-s001.zip › foods-1599056-supplementary.pdf]

foods

MDPI

Supplementary material

Table S1: Table of sensory attributes and their definitions for the profiling of yoghurts.

Sensory attributes	Definitions of attributes	Intensity range
	Odor	
**Sweet**	Basic odor quality which does not require definition	none - very intensive
**Sour**	Basic odor quality which does not require definition	none - very intensive
**Milky**	Characteristic milk odor	none - very intensive
**Yoghurt**	Characteristic yoghurt odor	none - very intensive
**Fat**	Characteristic fat milk odor	none - very intensive
**Green tea**	Characteristic green tea odor	none - very intensive
**Peach**	Characteristic odor of peach fruit	none - very intensive
**Nectar**	Characteristic odor of nectar and flowers	none - very intensive
**Citrus**	Characteristic odor of citrus fruits	none - very intensive
	Appearance perceived visually	
**Whey presence**	The presence of whey on the surface of the product and its quantity	none - a lot of
**Shine surface**	The appearance of gloss on the surface of the product	mat – shiny
**Colour intensity**	The color intensity of the product	light white - dark cream
**Visual smoothness**	The degree of smoothness of the product surface	rough – smooth
**Adhesiveness**	The density of the product measured by the resistance of the immersed teaspoon	thin – thick
**Filling the teaspoon**	Filling the spoon by placing the product on it	flat – conical
**Consistency uniformity**	Perception of product consistency uniformity	not uniform – uniform
	Texture/consistency perceived in the mouth	
**Thickness**	Perception of product density in the mouth	thin – thick
**Melting**	Perception of product melting in the mouth	much delayed – immediate
**Firmness**	Perception of product firmness in the mouth	loose – firm
**Yield stress**	Perception of product viscosity in the mouth	low viscosity- high viscosity
**Fat film**	Perception of a fatty film in the mouth	none - very intensive
**Creaminess**	Sensation of creaminess of the sample associated with melting	thin – creamy
**Smoothness**	Perception of product smoothness in the mouth	rough – smooth
	Taste/flavor	
**Sweet**	Basic taste quality which does not require definition	none - very intensive
**Sour**	Basic taste quality which does not require definition	none - very intensive
**Bitter**	Basic taste quality which does not require definition	none - very intensive
**Astringent**	Astringent sensation in the mouth	none - very intensive
**Milky**	Characteristic milk flavor	none - very intensive
**Yoghurt**	Characteristic yoghurt flavor	none - very intensive

**Quark**	Characteristic fresh cheese flavor	none - very intensive
**Green tea**	Characteristic green tea flavor	none - very intensive
**Peach**	Characteristic peach fruit flavor	none - very intensive
**Nectar**	Characteristic nectar/flowers flavor	none - very intensive
**Body**	Harmonisation of all positive attributes evaluated	incomplete – full
**Sensory overall quality**	Overall impression of harmony of the positive attributes presents with little or no negative attributes	low – high

Table S2: Results of sensory evaluation of yoghurts by panel of experts (n=20). The abbreviations in the table refer to the control sample (C), control yoghurt with 6% inulin (C1), yoghurt with green tea (G), yoghurt with green tea and 3% inulin (G1), yoghurt with green tea and 6% inulin (G2), yoghurt with green tea and 9% inulin (G3). (* significantly differed at $p \leq 0.05$).

	**C**	**C1**	**G**	**G1**	**G2**	**G3**
			**Odor**			
**Sweet***	1.0a	1.4ab	1.9bc	2.5bc	2.0c	2.7c
**Sour***	3.7bc	3.7c	2.6a	2.3a	2.8ab	2.6a
**Milky***	4.0c	3.9bc	3.2ab	2.9a	2.6a	2.7a
**Yoghurt***	4.2b	4.5b	3.3a	3.2a	3.0a	3.1a
**Fat**	3.0a	2.9a	2.8a	2.7a	2.7a	2.8a
**Green tea***	0.0a	0.0a	2.2b	1.4b	1.6b	1.7b
**Peach***	0.0a	0.1a	1.8b	2.8b	2.0b	2.7b
**Nectar***	0.1a	0.1a	1.0b	1.5b	1.0b	1.2b
**Citrus***	0.0a	0.0a	0.8b	0.9b	0.7b	0.8b
			**Appearance percived visually**			
**Whey presence***	3.1a	2.6a	5.7b	6.8c	7.8d	7.8d
**Shine**	7.3a	7.6a	7.3a	7.4a	7.5a	7.7a
**Colour ***	0.8a	0.9a	4.3b	4.2b	5.8c	5.6c
**Visually smoothness**	7.5a	7.3a	7.1a	7.8a	7.5a	7.6a
**Adhesiveness**	6.5a	7.0ab	6.9ab	7.0ab	7.5b	7.3b
**Teaspoon filling**	7.3a	7.5a	7.4a	7.1a	7.5a	7.6a
**Consistency uniformity**	7.5a	7.2a	6.9a	7.3a	7.4a	7.6a
			**Texture/Consistency perceived in the mouth**			
**Thickness in the mouth**	6.1a	6.5a	6.5a	6.3a	6.5a	6.7a
**Melting**	7.2a	6.7a	6.6a	6.3a	6.3a	6.5a
**Firmness**	6.1a	6.6abc	6.2ab	6.4abc	6.9bc	7.0c
**Yield stress***	2.2a	3.5c	2.9abc	2.7ab	3.1bc	2.8abc
**Fat film**	2.8ab	2.5ab	2.5ab	3.0ab	3.2b	2.4a
**Creaminess**	5.4a	5.7a	5.6a	5.7a	5.7a	5.6a
**Smoothness in the mouth**	7.3a	6.9a	7.2a	6.9a	7.2a	7.3a
			**Taste/ flavor**			
**Sweet***	1.9bc	4.0d	0.8a	1.6ab	1.9bc	2.5c
**Sour**	4.4bc	3.2a	4.7c	4.0abc	3.7abc	3.4ab
**Bitter***	0.4a	0.4a	1.9b	2.0b	2.4b	2.1b
**Astringent***	0.8a	0.4a	2.4b	2.1b	2.1b	2.1b
**Milky***	3.9b	3.8b	2.3a	2.5a	2.8a	2.8a
**Yoghurt***	5.1b	4.7b	2.9a	3.5a	3.2a	3.3a

**Quark**	2.2a	2.5a	2.1a	2.4a	1.6a	1.8a
**Green tea***	0.0a	0.0a	2.7b	2.5b	2.3b	1.9b
**Peach***	0.0a	0.0a	0.9b	1.9c	1.5bc	1.9c
**Nectar***	0.0a	0.0a	0.5ab	0.7b	0.7b	0.7b
**Body***	5.9b	5.9b	5.2ab	4.5a	4.9a	5.4b
**Sensory overall quality***	6.4c	6.5c	5.1ab	4.7a	5.1ab	5.6bc

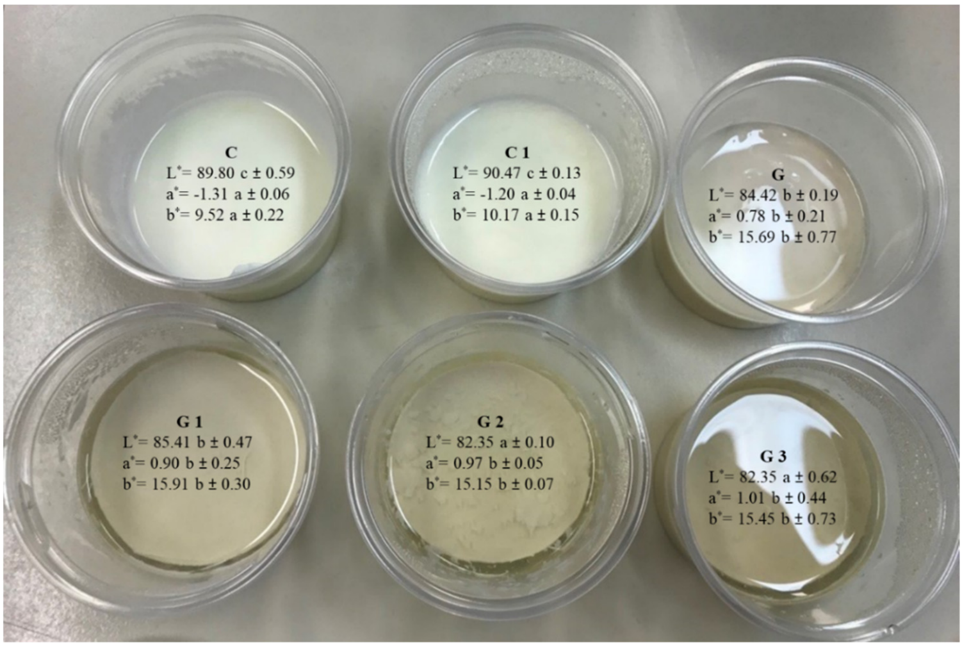

Figure S1: Colour parameters in yoghurts: C, C1, G, G1, G2, G3. The abbreviations in the figure refer to the control sample (C), control yoghurt with 6% inulin (C1), yoghurt with green tea (G), yoghurt with green tea and 3% inulin (G1), yoghurt with green tea and 6% inulin (G2), yoghurt with green tea and 9% inulin (G3).
